# Characteristics and sources of atmospheric pollutants in typical inland cities in arid regions of central Asia: A case study of Urumqi city

**DOI:** 10.1371/journal.pone.0249563

**Published:** 2021-04-20

**Authors:** Zongying Li, Yao Wang, Zhonglin Xu, Yue’e Cao

**Affiliations:** 1 College of Resource and Environmental Science, Xinjiang University, Urumqi, China; 2 Key Laboratory of Oasis Ecology, Ministry of Education, Urumqi, China; 3 Institute of Desert Meteorology, China Meteorological Administration, Urumqi, China; 4 School of Environmental and Geographical Sciences, Shanghai Normal University, Shanghai, China; Universidade de Vigo, SPAIN

## Abstract

The arid zone of central Asia secluded inland and has the typical features of the atmosphere. Human activities have had a significant impact on the air quality in this region. Urumqi is a key city in the core area of the Silk Road and an important economic center in Northwestern China. The urban environment is playing an increasingly important role in regional development. To study the characteristics and influencing factors of the main atmospheric pollutants in Urumqi, this study selected Urumqi’s daily air quality index (AQI) data and observation data of six major pollutants including fine particulate matter (PM_2.5_), breathable particulate matter (PM_10_), sulfur dioxide (SO_2_), nitrogen dioxide (NO_2_), carbon monoxide (CO), and ozone (O_3_8h_) from 2014 to 2018 in conjunction with meteorological data to use a backward trajectory analysis method to study the main characteristics of atmospheric pollutants and their sources in Urumqi from 2014 to 2018. The results showed that: (1) From 2014 to 2018, the annual average of PM_2.5_, PM_10_, SO_2_, NO_2_ and CO concentrations showed a downward trend, and O_3_8h_ concentrations first increased, then decreased, and then increased, reaching the highest value in 2018 (82.15 μg·m^-3^); The seasonal changes of PM_2.5_, PM_10_, SO_2_, NO_2_ and CO concentrations were characterized by low values in summer and fall seasons and high values in winter and spring seasons. The concentration of O_3_8h_, however, was in the opposite trend, showing the high values in summer and fall seasons, and low values in winter and spring seasons. From 2014 to 2018, with the exception of O_3_8h_, the concentration changes of the other five major air pollutants were high in December, January, and February, and low in May, June, and July; the daily changes showed a “U-shaped” change during the year. The high-value areas of the "U-shaped" mode formed around the 50th day and the 350th day. (2) The high-value area of AQI was from the end of fall (November) to the beginning of the following spring (March), and the low-value area was from April to October. It showed a U-shaped change trend during the year and the value was mainly distributed between 50 and 100. (3) The concentrations of major air pollutants in Urumqi were significantly negatively correlated with precipitation, temperature, and humidity (*P*<0.01), and had the highest correlation coefficients with temperature. (4) Based on the above analysis results, this study analyzed two severe pollution events from late November to early December. Analysis showed that the PM_2.5_/PM_10_ ratio in two events remained at about 0.1 when the pollution occurred, but was higher before and after the pollution (up to 1.46). It was shown that the pollution was a simple sandstorm process. Backward trajectory analysis clustered the airflow trajectories reaching Urumqi into 4 categories, and the trajectories from central Asia contributed the maximum values of average PM_2.5_ and PM_10_ concentrations.

## 1 Introduction

Since the political and economic restructuring and opening up, China’s urbanization level has increased from 18% in the early stages(1980s’) to the current of 57.40% [1,2], and the number of urban populations has increased from 173 million in 1979 to 813 million in 2017 [3]. The development of China’s urbanization started late [1] and was mainly followed a partial and short-term targeted developing pattern in the early stage [4]. The ecological protection and environmental governance work left behind, which has caused a series of ecological and environmental problems [5,6]. Among these environmental problems, the air pollution in urban areas is one of the most serious ones. Since the appearance of the air pollution, there have been a large number of related studies try to explain the internal mechanism of air quality changes and provide feasible guidance and related suggestions for improving urban air quality [7–10].

In China, urban air pollution in different urban areas has different properties, but the coarse particulate matter (PM_10_) and fine particulate matter (PM_2.5_) are the two pollutants that have been paid more attention than others (such as sulfur dioxide and nitrogen dioxide) by scientists. It has been proved that the air quality in Chinese cities was generally improved, and the proportion of cities that meet air quality standards is rising [11]. The Beijing-Tianjin-Hebei region (three provinces around Beijing, also named Jingjinji region) was classified as the poorest air quality region in China and corresponding research have shown that high PM_2.5_ concentration of the region was due to coal consumption, high population density and construction [12]. Based on analysis of the source of air pollution, two transmission paths of pollutants has been identified by Gao, Wang [13] and they also found that the presence of a pressure equalization field, a low-level inversion layer, and the southern warm and humid airflow provided favorable conditions for the formation of PM_2.5_ in the region. The central plain region and Yangtze River Delta area of China are also facing the problem of air pollution mainly contribute by PM_10_ and PM_2.5_, as has been reported elsewhere [14,15].

The current studies corresponding to air quality of urban area in China mainly focused PM_10_ and PM_2.5_ and conducted in eastern coastal areas or central plain region of China, little attention has been paid in urban area of northwestern part (also inland part) of the country. It should be noted that the urbanization in inland regions also witnessed severe air pollution [16], a systematic analysis on the component, source and transmission path of urban air pollution is helpful to provide decision support for the government to make reasonable and effective air pollution control measures in these regions. This study took the Urumqi city (in recent years, the air pollution has become increasingly prominent in urban areas of the city) in northwest part of China as an example to analyze the spatial-temporal characteristics of air pollution of typical inland city in northwest China. Specifically, we aimed to understanding the component, source and transmission path of the pollutant of the city and quantify the relationship between the Air Quality Index (AQI) with meteorological data based on air quality observation data collected from 2014 to 2018. The backward trajectory analysis was used to study the temporal variation of two severe and typical air pollution events that occurred from late November to early December in 2018.

## 2. Materials and methods

### 2.1 Study area

**Fig 1:**
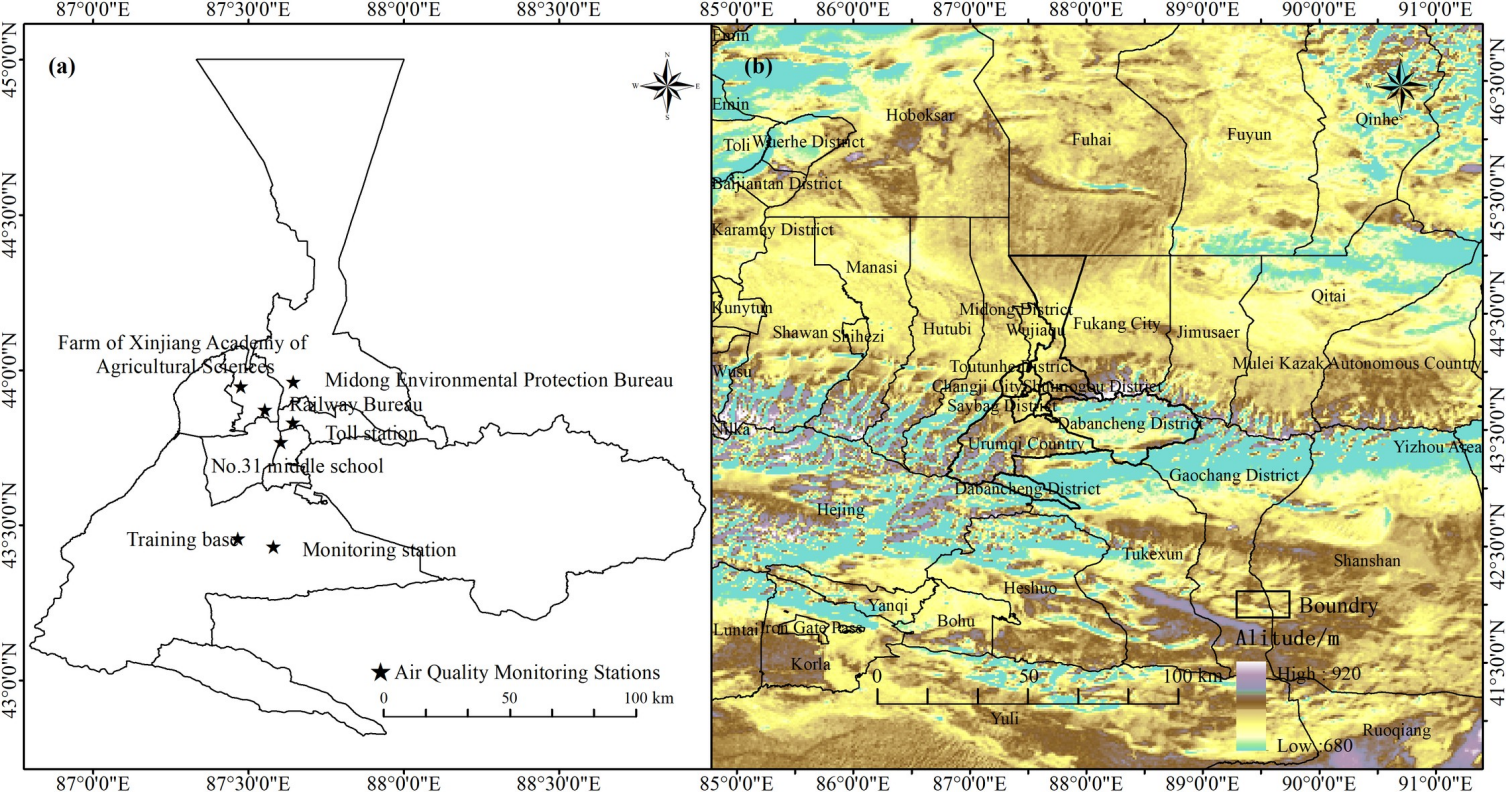
Map of study area ((a)Location and distribution of monitoring stations; (b)Orographic distribution in Urumqi and its surrounding areas).

Urumqi (42°45′32″-44°08′00″N, 86°37′33″-88°58′24″E, total area of 14,216 km^2^), locating in the hinterland of Eurasia and is one of the most important cities of the Silk Road (Fig 1A). The city is experiencing rapid economic development and population growth at the present. The GDP reached 309.98 billion RMB, an increase of 7.8% over the previous year in 2018. According to statistics yearbook 2018, Urumqi reached 90.2% of urbanization rate and a build-up area of 365.88 km^2^. Urumqi is surrounded by mountains in the east, south and west, with high terrain in the southeast and low in the northwest (Fig 1B). The elevation of the city ranges from 680 to 920 meters with an average of 800 meters in the urban area. It has a temperate continental arid climate. The mean temperature is 7.3°C and reached the highest in July and August with an average of 25.7°C, and lowest in January with an average of -15.2°C. The annual average precipitation is 236 mm, and the 64% falls in Spring and Summer.

### 2.2 Data sources

The AQI is a dimensionless indicator that quantitatively describes the air quality [17–20]. According to new ambient air quality standard of China (GB395-2012), the index is the highest concentrations of PM_2.5_, PM_10_, sulfur dioxide (SO_2_), nitrogen dioxide (NO_2_), carbon monoxide (CO), and maximum 8-hour average ozone (O_3_8h_). There are also different definition of AQI, such as fuzzy-based AQI (FAQI), which weighted the concentration of PM_10_, SO_2_, NO_2_, CO and O_3_8h_, based on fuzzy algorithm [21] and air pollution index (API), which do not cover the concentration of PM_2.5_ [22]. The present study considered the AQI and the level of classification are shown in Table 1. AQI is widely used in China due to its simple definition [23,24].

**Table 1:** Standard of air quality index [24,25] (GB3095-2012 environmental air quality standard).

AQI	Air Quality Level	Air Quality Grade	Health Effects
0–50	Level 1	Excellent	The body feels comfortable
51–100	Level 2	Good	A very small number of very sensitive people feel lightly ill
101–150	Level 3	Light pollution	People susceptible to increased discomfort, healthy people appear stimulating symptoms
151–200	Level 4	Moderate pollution	Exacerbates symptoms in susceptible populations and causes respiratory distress in healthy populations
201–300	Level 5	Heavy pollution	Patients with heart and lung disease are uncomfortable and exercise tolerance is reduced
>300	Level 6	Severe pollution	Exercise tolerance is reduced and early symptoms of some diseases appear among healthy people

The air quality monitoring data (concentrations of PM_2.5_, PM_10_, SO_2_, NO_2_, CO and O_3_8h_) used to calculate the AQI in this study was obtained from the China Air Quality Monitoring and Analysis Platform (https://www.aqistudy.cn/). Seven automatic air quality monitoring stations (Training base, Toll station, Monitoring station, No.31 middle school, Railway Bureau, Farm of Xinjiang Academy of Agricultural Sciences, Midong Environmental Protection Bureau) were selected. These seven air quality monitoring stations were classified according to functional areas, representing clean areas (Training base and Farm of Xinjiang Academy of Agricultural Sciences), residential areas (Toll station, Monitoring station and Railway Bureau), industrial areas (Midong District Environmental Protection Bureau), and cultural and educational areas (No.31 Middle School). When calculating the AQI, we used the daily average of these seven monitoring stations in Urumqi. The data was collected from January 1, 2014 to December 31, 2018. Meteorological data for backward trajectory analysis were downloaded from synchronized Global Data Assimilation System (GDAS) maintained by the National Center for Environmental Forecasting (NCEP, ftp://arlftp.arlhq.noaa.gov/pub/archives/gdas1). The climate data were obtained from China Meteorological Data Network (http://data.cma.cn/).

China Air Quality Monitoring and Analysis Platform is an automatic air quality monitoring system consists of monitoring stations, quality control laboratories and system support laboratories. The quality control laboratories are responsible for the standardization, calibration and audit of monitoring equipment, ensuring the accurate transmission and storage of data. In addition, these laboratories also responsible for correcting and eliminating of the abnormal data (missing data and errors caused by non-human factors such as instrument failure and power failure). With the help of the laboratory, the system could provide accurate, reliable, continuous and timely environmental monitoring data including AQI, PM_2.5_, PM_10_, SO_2_, NO_2_, O_3_, CO, temperature, humidity, wind force scale, wind direction, satellite cloud image and other monitoring items. In the system, all data are updated hourly and automatically.

### 2.3 Statistical analysis

In this study, the Pearson’s correlation coefficient was used to analyze the relationship between AQI and the meteorological factors, the simple liner regression was used to quantify the response of AQI to meteorological factors, the corresponding calculations were performed use the Statistics Package for Social Science software v.22.0 (SPSS Inc, Chicago, IL). In addition, the MeteoInfo [26] software was used to analyze the backward trajectory and related statistical analysis.

TrajStat trajectory model, a plug-in of MeteoInfo, is used for backward trajectory analysis, which is a professional model for calculating air mass trajectory and was widely used in the study of air pollutant transport and diffusion. In this paper, we analyzed the backward trajectory of the air flow that affected two air pollution events in Urumqi (the first one appeared in November 25^th^ 2018 and the second one started at 1^st^ and ended at 2^nd^ in December of 2018). The corresponding analysis was simulated at height of 500 m from the ground and the center of Urumqi (43°37′N, 87°50′E) were setup as starting point. In addition, the trajectory estimation time is 24h. Based on the simulation results, the air flow trajectories gathered in Urumqi were classified into four categories. Furthermore, the different transport trajectories of PM_2.5_ and PM_10_ are obtained by overlying the concentrations of PM_2.5_ and PM_10_ to the classified categories.

## 3. Results

### 3.1 Temporal variation of major atmospheric pollutants

**Fig 2:**
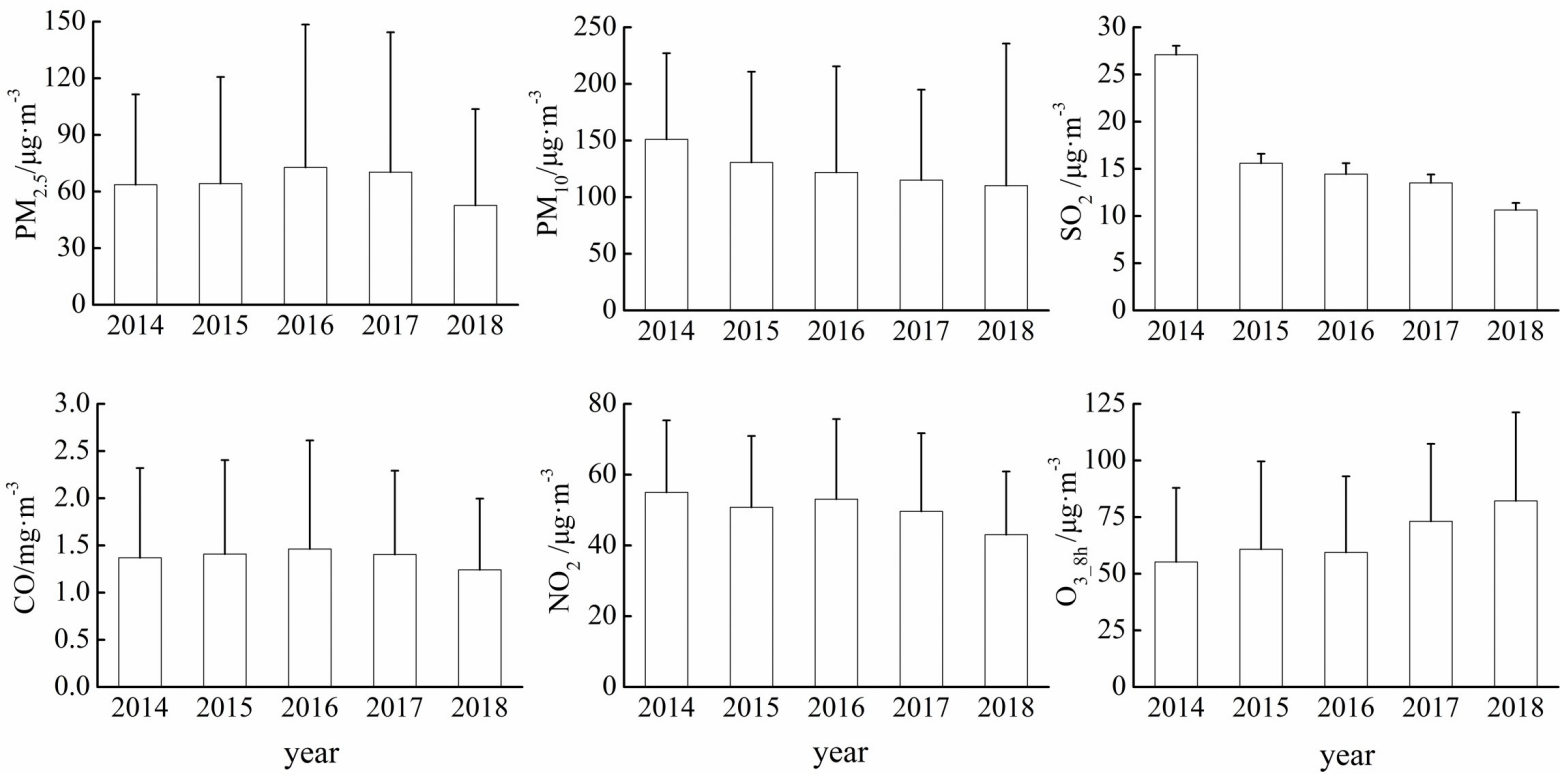
Inter-annual variations of major air pollutants in Urumqi.

From 2014 to 2018, the annual average concentrations of PM_2.5_ and CO followed an increase-decrease trend and reached the highest values (72.77 μg·m^-3^ and 1.46 mg·m^-3^, respectively) in 2016 and the lowest values (52.64 μg·m^-3^ and 1.24 mg·m^-3^, respectively) in 2018 (Fig 2). The concentrations of PM_10_ and SO_2_ showed a downward trend and reached the lowest values (110.15 μg·m^-3^and 10.64 μg·m^-3^, respectively) in 2018. The inter-annual variation of NO_2_ concentration fluctuated from 2014 to 2018 and the highest (54.99 μg·m^-3^) and the lowest (43.50 μg·m^-3^) concentrations of NO_2_ appeared in 2014 and 2018, respectively. The concentration of O_3_8h_ showed an upward trend, which declined slightly in 2016 and reached the highest value (82.15 μg·m^-3^) in 2018.

**Fig 3:**
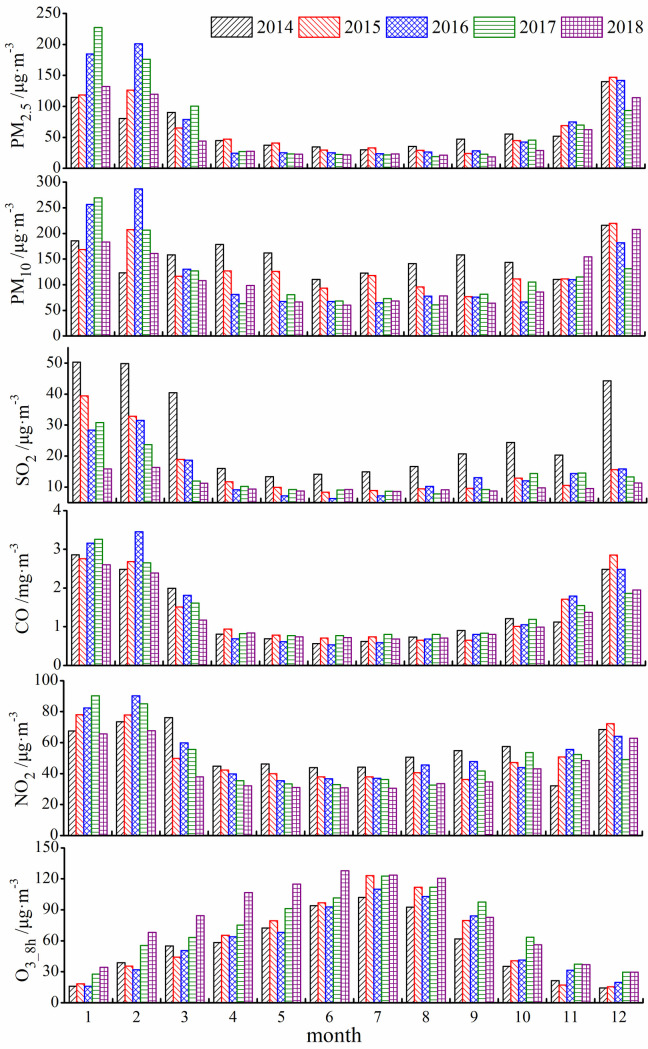
Monthly changes of major air pollutants in Urumqi.

The inter-annual variations of PM_2.5_, PM_10_, SO_2_, NO_2_ and CO were similar with lowest and highest value appeared in summer and winter, respectively (Fig 3). In contrast, the concentration changes of O_3_8h_ reached highest in summer and lowest in winter. The Urumqi is the remotest city from any ocean in the word and the feather of climate is short warm summers and long cold winters [27]. It determined that Urumqi has very high demands and consumption rates of fossil fuels for wintertime heating. Moreover, lots of industries and people were attracted to Urumqi from all parts of China with the strongly developing economy, enhanced the increasing consumption of energy by fossil fuels and the steady growing fleet of motor vehicles. It is likely to explain the characteristics of pollutants (PM_2.5_, PM_10_, SO_2_, NO_2_ and CO) mentioned above in this area. The variations concentration of O_3_8h_ rather different from other pollutants, as well, it can be explained by the increasing of solar radiation [28].

The monthly average concentrations of PM_2.5_ and CO were low from April to September and started to rise in October, after reached the annual highest in December and January, they gradually decreased from February to March (Fig 3). During the study period (from January, 2014 to December, 2018), monthly concentration of PM_2.5_ reached its highest value (227.52 μg·m^-3^) in January, 2017. For CO, the highest concentration value (3.45 mg·m^-3^) were reached in February 2016 (Fig 3). In February 2016, the concentrations of PM_10_ reached its monthly highest value in five years with 286.69 μg·m^-3^. The monthly average concentration of SO_2_ was obviously higher in 2014 than in other years (especially in December). NO_2_ concentration reached its highest value (90.19 μg·m^-3^) in January 2017. The monthly average concentration of O_3_8h_ followed a unimodal pattern in all years and reached the highest value in July. Compare with other years, the O_3_8h_ concentration was higher in 2018.

**Fig 4:**
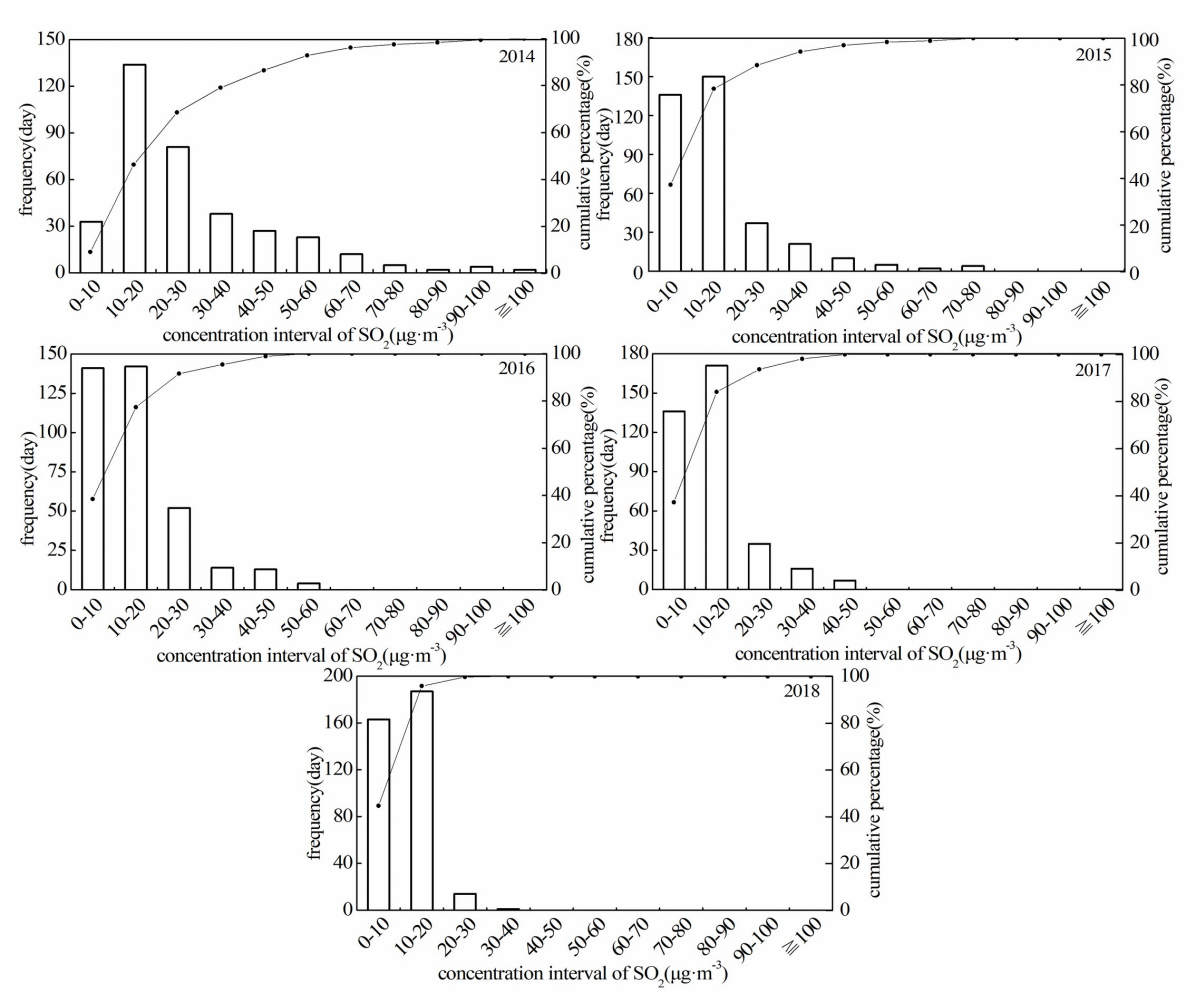
Histogram of daily SO_2_ concentration distribution in Urumqi.

During our study period, the interannual and seasonal variations of PM_2.5_, PM_10_, SO_2_, CO, and NO_2_ concentrations showed a decreasing trend, and the variation of SO_2_ concentrations was the most obviously (Figs 2 and 3). Additionally, the histogram (Fig 4) demonstrated that the variation range of SO_2_ concentration is shrinking, decreasing yearly and the concentration tends to be stable (*CV* from 70.53% in 2014 to 36.96% in 2018, Table 2). It should be noted that coal combustion is one of the main sources of urban air pollution in most of cities at western of China [29,30]. In 2014, aimed to reduce the degree of air pollution and improve the quality of human settlements, Urumqi launched 22 projects including energy conservation, air pollution prevention, and water pollution prevention, etc. In terms of air pollution control, the corresponding projects including coal-to-gas (a measure to change the energy structure), grid-connected boiler network (compare to independent heating unit) and relocation of polluting enterprises [31]. Due to these effective measures (along with air pollution prevention measures implemented in the steel industries and enhancement of transformation and obsolescence of high-emission equipment), the concentration of SO_2_ significantly declined from 2014 to 2018, as shows in the present study and elsewhere [32]. Additionally, the meteorological condition also contributed to the high concentration of SO_2_, since when temperature is low, an inversion layer is easy to generate for a city located in valley, and the layer could hinder the diffusion of pollutants [33]. It is important to note although the concentrations of PM_2.5_, PM_10_, CO, NO_2_ also decreased, the reduction is less effective compare with SO_2_. This might be due to the measures (primarily aimed at reducing pollutants emitted by industry and energy use) that currently the local government enacted [32]. Since the air pollution is dominated by particulate pollutants (PM_2.5_ and PM_10_), more effective prevention and control measures for particulate pollutants would fundamentally improve air quality in the region.

**Table 2:** The Statistical characteristics of variation of main pollutant concentrations in Urumqi.

	**PM**_**2.5**_**/μg·m**^**-3**^			**PM**_**10**_**/μg·m**^**-3**^			**SO**_**2**_**/μg·m**^**-3**^		
	**Mean**	**Standard Deviation**	**Variable coefficient (*CV*/%)**	**Mean**	**Standard Deviation**	**Variable coefficient (*CV*/%)**	**Mean**	**Standard Deviation**	**Variable coefficient (*CV*/%)**
2014	63.62	47.87	**75.25**	151.00	76.14	**50.42**	27.10	19.11	**70.53**
2015	64.26	56.45	**87.86**	130.65	80.14	**61.34**	15.60	12.56	**80.55**
2016	72.77	75.69	**104.02**	121.88	93.57	**76.77**	14.45	10.26	**70.98**
2017	70.31	74.09	**105.38**	115.05	79.83	**69.39**	13.52	7.97	**58.98**
2018	52.64	51.02	**96.93**	110.15	125.48	**113.92**	10.64	3.93	**36.96**
	**CO/mg·m**^**-3**^			**NO**_**2**_**/μg·m**^**-3**^			**O**_**3**_**_8h/μg·m**^**-3**^		
	**Mean**	**Standard Deviation**	**Variable coefficient (*CV*/%)**	**Mean**	**Standard Deviation**	**Variable coefficient (*CV*/%)**	**Mean**	**Standard Deviation**	**Variable coefficient (*CV*/%)**
2014	1.37	0.95	**69.29**	54.99	20.34	**36.98**	55.19	32.68	**59.22**
2015	1.41	1.00	**70.72**	50.76	20.16	**39.71**	60.80	38.78	**63.79**
2016	1.46	1.15	**78.55**	53.08	22.63	**42.62**	59.43	33.59	**56.53**
2017	1.40	0.89	**63.19**	49.64	22.06	**44.45**	73.13	34.20	**46.76**
2018	1.24	0.75	**60.78**	43.05	17.85	**41.45**	82.15	39.10	**47.59**

**Fig 5:**
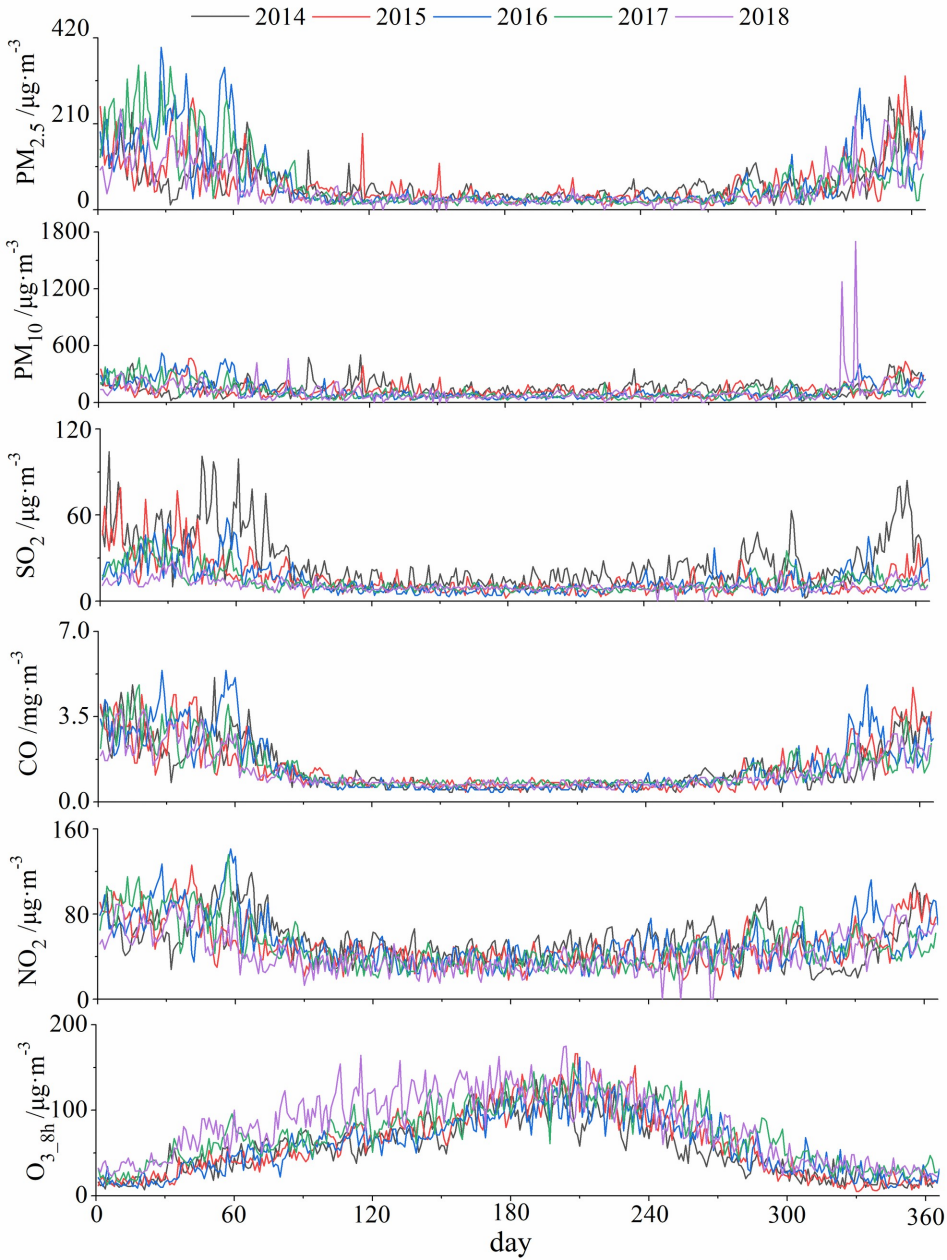
Daily changes of atmospheric pollutants in Urumqi.

The daily concentration of PM_2.5_, PM_10_, SO_2_, NO_2_, CO and O_3_8h_ were similar with their intra-annual variation (Fig 5). Among these six pollutants, PM_2.5_ and CO varied synchronously throughout the entire study period. Interestingly, for concentrations of PM_2.5_, PM_10_, SO_2_, and CO, the lower (higher) the concentrations itself, the lower (higher) the standard deviation of the concentrations. In contrast, the NO_2_ and O_3_8h_ did not displayed such pattern. In addition, there exist two extreme concentrations of PM_10_ in late November and early December of 2018 (1274 μg·m^-3^ and 1700 μg·m^-3^), indicating that there was a serious air pollution event in Urumqi during the period.

### 3.2 variation of in AQI

**Fig 6:**
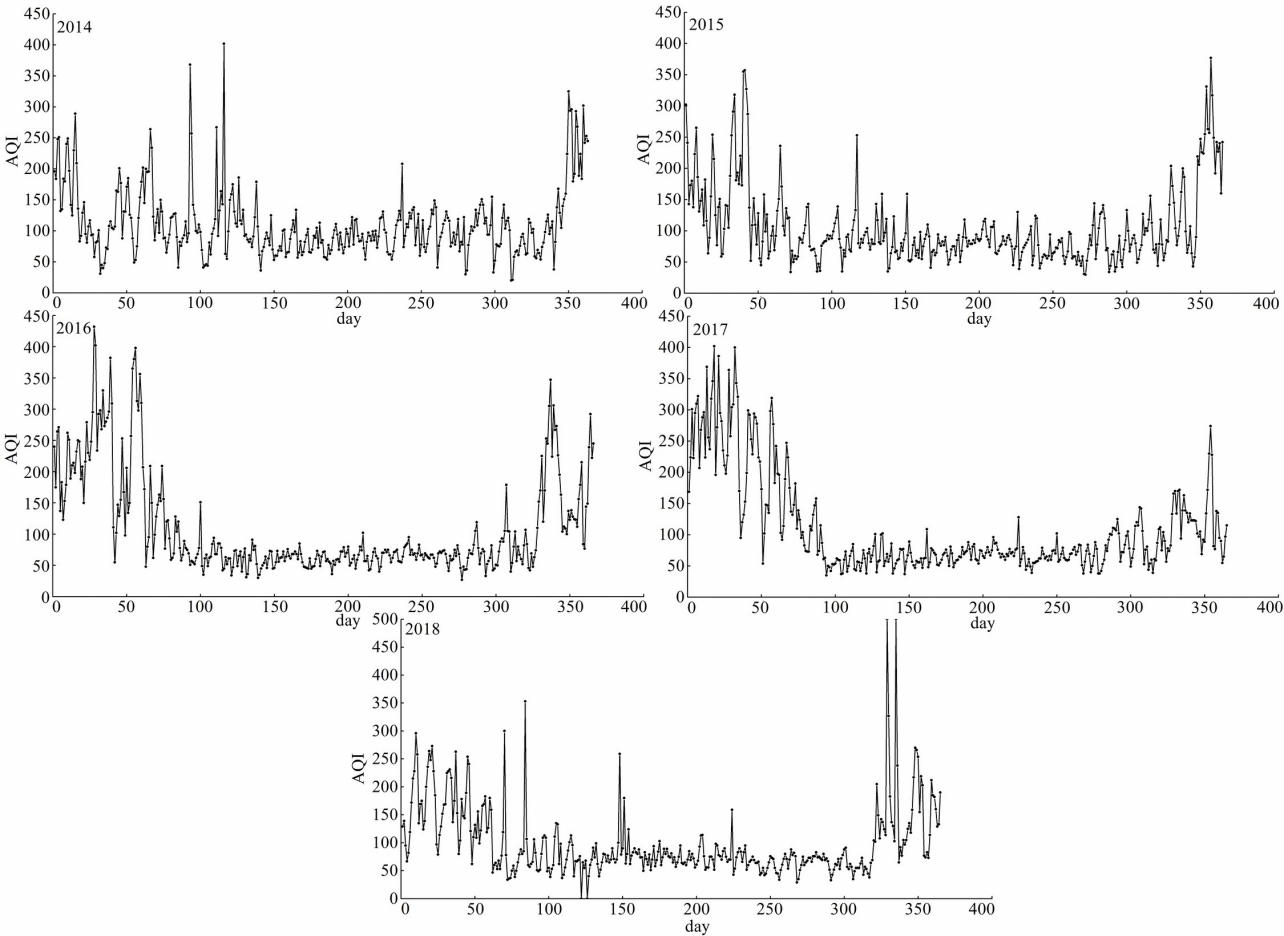
Daily changes of AQI in Urumqi.

**Fig 7:**
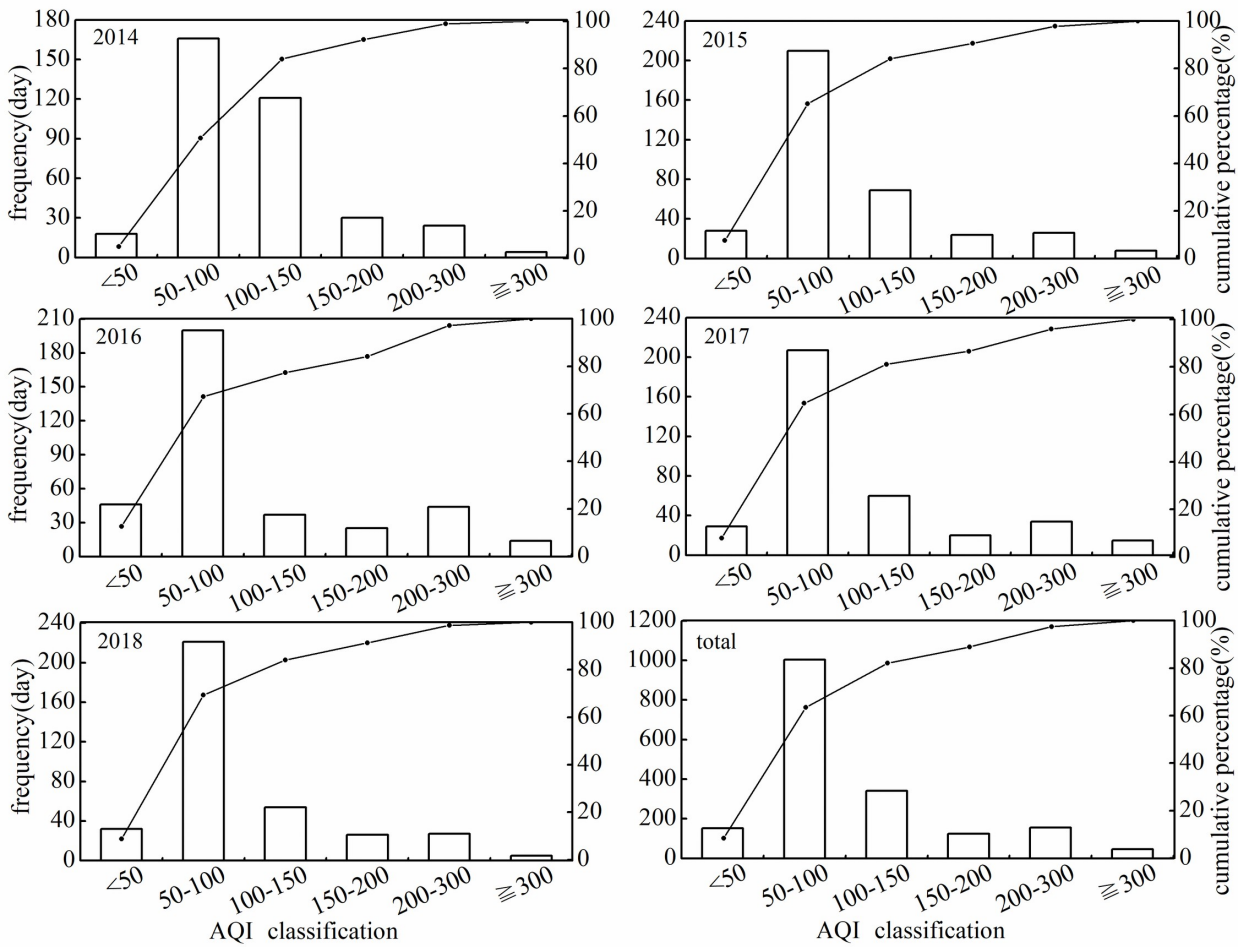
Frequency of pollution levels and cumulative percentage changes.

AQI was relatively higher from November of first year to March of the following year and lower from April to October with a unimodal trend. Majority of AQI value ranged between 51–100, which denoted good air quality (Fig 6). The proportion of days with good air quality (AQI value ranged between 51–100) was 45.73% in 2014 and increased to 60.55% in 2018 (Fig 7). Overall, number of days with excellent air quality (AQI value less than 51) increased from 2014 to 2018. Moreover, number of days with severe air quality (AQI value higher than 300) was fewest among six air quality levels. The light, moderate and heavy pollution (the range of AQI value are 101–150, 151–200 and 201–300, respectively) days may appear in any time of the year, but the possibility was relatively higher from November to May of next year. Summer (May to August) was the optimal season with the best air quality and winter (December to February) was the worst one.

### 3.3 Analysis of meteorological factors

As displays in Table 3, among six meteorological factors, the temperature is the only factor that have relatively higher correlation coefficients with six pollutants. Specifically, it significantly and negatively correlated with PM_2.5_, PM_10_, SO_2_, CO, and NO_2_ (*p*<0.01), and significantly and positively correlated with O_3_8h_ (*p*<0.01). The correlation coefficient between humidity, air pressure and wind speed with six pollutants are less than 0.3, which indicated that they had little effect on the concentration of pollutants.

**Table 3:** Correlation coefficients between main pollutants and meteorological factors in Urumqi.

Pollutants	Precipitation	Temperature	Humidity	Air Pressure	Wind Speed
PM_2.5_	-0.212**	-0.555**	-0.170**	-0.003	-0.025
PM_10_	-0.201**	-0.299**	-0.300**	-0.011	0.094**
SO_2_	-0.193**	-0.430**	-0.183**	-0.032	-0.038
CO	-0.216**	-0.659**	-0.116**	0.030	-0.067**
NO_2_	-0.291**	-0.489**	-0.261**	0.035	0.016
O_3__8h	0.101**	0.812**	-0.221**	-0.187**	0.209**

**significant with *p*<0.01

* significant with *p*<0.05.

### 3.4 Analysis of two severe pollution events in late November and early December of 2018

**Fig 8:**
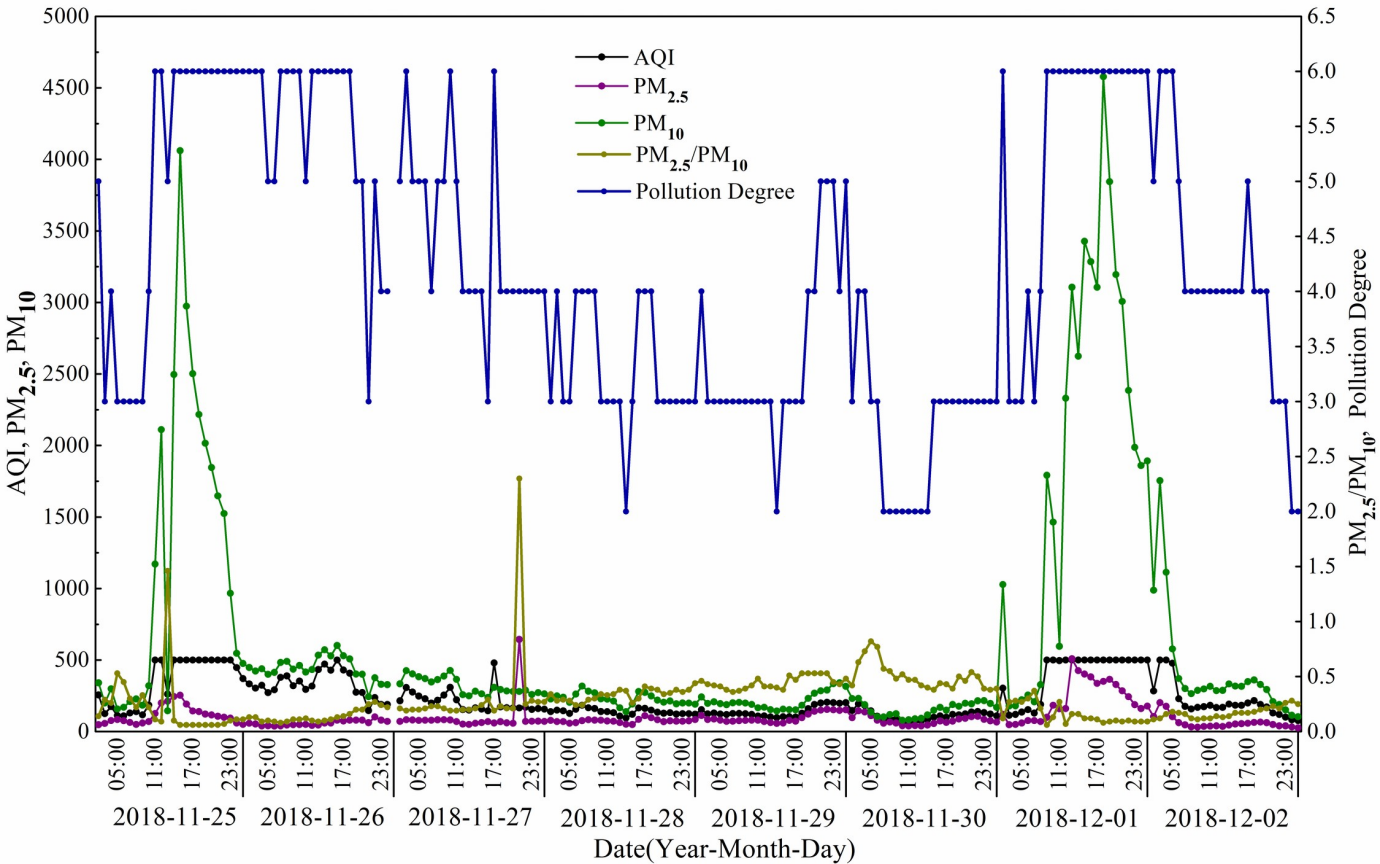
Variations of AQI and concentrations of PM_2.5_ and PM_10_ during the two air pollution events emerged from November 25^th^ to December 2^nd^, 2018.

From November 25^th^ to December 2^nd^, 2018, Urumqi suffered two separate severe air pollution events (Fig 8). During the two events, the AQI is maintained at the highest value of 500 and the pollution level is 6, which are serious pollution events. The first one began at 9:00 on November 25^th^, 2018 and ended at 20:00 on the same day. Within 12 hours of the event, the concentrations of PM_2.5_ and PM_10_ exceeded 100 μg·m^-3^ and 1000 μg·m^-3^, respectively, and reached their highest value with 254 μg·m^-3^ and 4061 μg·m^-3^. The concentrations of PM_2.5_ and PM_10_ decreased rapidly to normal values in 6 hours after the event ended. The second event lasted 21 hours and the concentrations of PM_2.5_ and PM_10_ reached 120 μg·m^-3^ and 1028 μg·m^-3^, respectively, at 0:00 of December 1^st^, and then returned to normal values. The pollution restarted at 7:00 and concentrations of PM_2.5_ and PM_10_ rose to the highest values with 351 μg·m^-3^ and 4578 μg·m^-3^, respectively, at 16:00. The PM_2.5_/PM_10_ ratio maintained around 0.1 during the two events and increased to about 1.46 after the second event, indicated that the pollutants of the two events dominated by sandstorm.

**Fig 9:**
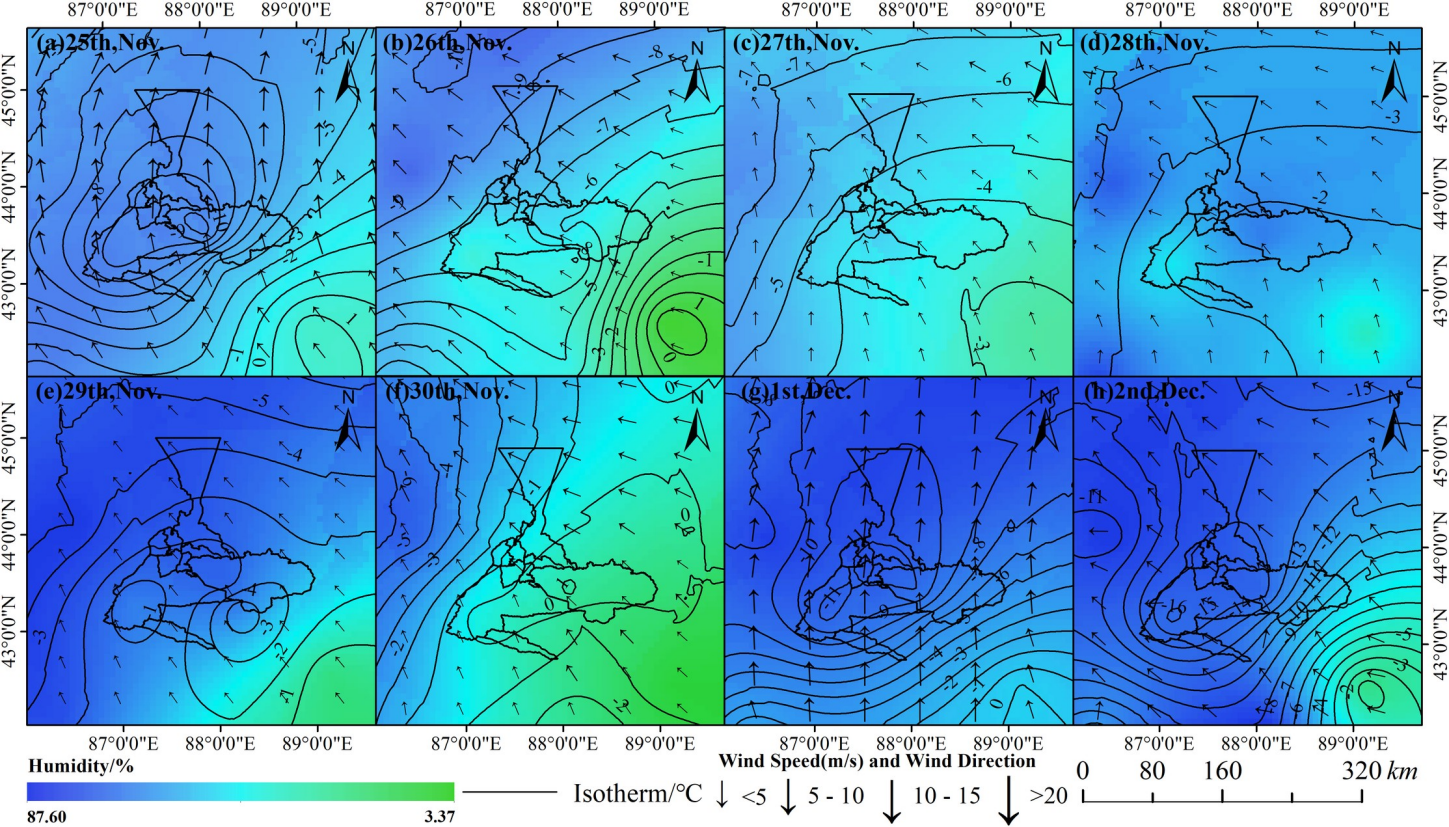
Weather conditions in Urumqi and its surrounding areas from November 25^th^ to December 2^nd^, 2018.

The two air pollution events mainly occurred on November 25^th^, and from December 1^st^ to 2^nd^, 2018, the characteristics of the local weather was air humidity increased significantly, accompanied by precipitation and strong winds. The meteorological conditions accompanied pollution events mainly show the following characteristics (Fig 9): Firstly, the relative air humidity during the events was apparently higher than the humidity before and after pollution, and the spatial distribution of relative humidity is higher in northwest than in southeast portion of the study area. Secondly, the isotherm is more concentrated and curved during the events (Fig 9A, 9G and 9H) compare with other time (Fig 9B–9F), indicating the drastic variation of air temperature. Finally, during the events, the wind speed is faster (the maximum wind speed is more than 10m/s), and the wind direction was also changed (dominated by south and southeast wind).

**Fig 10:**
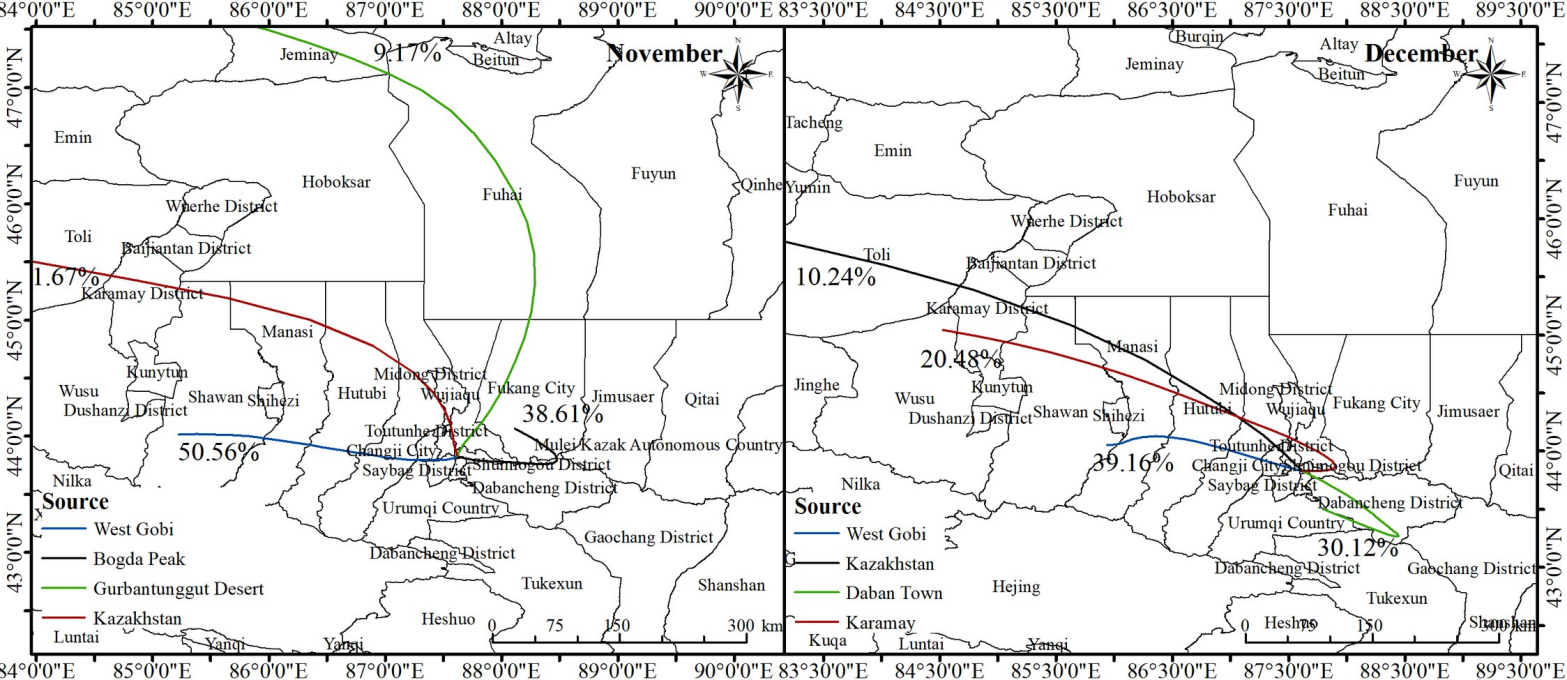
Backward trajectory cluster distribution.

Backward trajectories analysis revealed that air mass trajectories that arrived in Urumqi could be classified to four categories with the major source originated from west and southeast, and the transmission paths is consistent with the wind direction (Fig 10). The trajectory with the largest number of airflows originated from the Gobi Desert in the west of Urumqi. The transmission distance was short and the overall airflow of the Gobi Desert accounting for 50.56% and 39.16% of the total flows during the two events, respectively. The trajectory with the longest transmission distance came from Central Asia (Kazakhstan) and brought sandy pollutants from the Gurban Tungut Desert. This trajectory accounted for 1.67% and 10.24% of the total airflow in the first and second event, respectively.

The distribution of various trajectories, the concentrations of corresponding pollutants, and terrain around Urumqi demonstrated that (Table 4 and Fig 1B): (1) The trajectory from Central Asia Kazakhstan had a long transmission distance and came from the sand source area. There were enough sand and dust pollutants and active atmospheric reaction conditions. The concentrations of PM_2.5_ and PM_10_ were the highest. (2) The trajectories from the West Gobi and Daban Town were easy to accumulate pollutants due to the short transmission distance and relatively stable meteorological conditions. (3) Compared with the air mass trajectory in December, the trajectory from the Karamay was close to and intersected with the trajectory from Central Asia in December. The concentrations of PM_2.5_ and PM_10_ were reached 81.82 μg·m^-3^ and 555.50 μg·m^-3^, respectively. (4) The minimums of PM_2.5_ and PM_10_ occurred in November in the Central Asian airflow trajectory because of the long transmission distance and obstruction of the Bogda Peak, reached 20.12 μg·m^-3^ and 23.15 μg·m^-3^, respectively.

**Table 4:** Distribution characteristics and average pollutant concentration of all backward trajectory.

Time	Clustering	Source	Path Area	Probability%	PM_2.5_ Concentration (μg/m^-3^)	PM_10_ Concentration (μg/m^-3^)
Nov.	1	West of China	West Gobi, Changji	50.56	80.98	347.54
	2	East of China	Bogda Peak, Daban T own	38.61	48.24	337.05
	3	North of China	Kazakhstan, Ulungur Lake, Gurbantunggut Desert, Fukang,	9.17	20.12	23.15
	4	West of China	Kazakhstan, Tacheng area, Karamay, Gurbantunggut Desert	1.67	142.58	1384.47
Dec.	1	West of China	West Gobi, South of Shihezi, Hutubi, Urumqi	39.16	55.02	147.66
	2	Northwest of China	Kazakhstan, Tacheng area, Karamay, Gurbantunggut Desert	10.24	237.65	2031.76
	3	Southeast of China	urumqi county, Daban Town	30.12	73.10	105.66
	4	Northwest of China	Karamay, North of Shihezi, Wujiaqu, Midong District	20.43	81.82	555.50

## 4. Discussion

Several studies have shown that meteorological conditions have great influence on the concentration of atmospheric pollutants in cities with similar climate like Urumqi [34–36]. In general, air pollution in these cities was prone to occur during heating systems are operating (beginning at November until the end of March) and high dust generation periods (usually the Spring), rather than periods with strong ultraviolet radiation [18]. This is confirmed by the high negative correlations between the concentrations of six major air pollutants and air temperature (Table 3). The intensively developed continental anticyclone control the climate of Urumqi, downward flows strengthened the accumulation of pollutants, and snow-covered surface results in strong cooling of the adjacent air, which in turn is prone for the build-up of surface inversions. As mentioned previously, an inversion layer which could hinder the diffusion of pollutants is easy to generate for a city located in a valley [33]. In contrast, the air quality in coastal cities is mainly affected by other climatic conditions such as the monsoon [37,38], humidity [39,40] or atmospheric pressure [41,42]. It is interesting that in our study, the correlation relationship between wind speed and concentrations of six major pollutants in Urumqi is weak (Table 3). This is different from Trabzon city [43], Florence, Milan and Vicence [44] where diffusion effect of wind usually results in a relatively high correlation coefficient between the concentration of atmospheric pollutant and wind speed. We speculate the weak relationship in Urumqi might be due to the balance of two mechanisms. Firstly, although in summer the wind speed is high, which is favorable condition to sand storm, whereas abundant rainfall in the same season restricted the floating of dust [45]. Secondly, a relatively lower wind speed in winter is accompanied by lower air temperature, which usually results an inversion layer that prevents the diffusion of pollutants [33].

The topographical situation played an important role in air pollution of the city according to the analysis of PM concentrations in Xinjiang [46,47], which contains China’s two large deserts, the Taklimakan and Gurbantunggut desert. Moreover, the west, east, and the south of Urumqi are bordered by Tianshan mountains, the north is resisted by northwest wind from Siberia carrying abundant dust (the northwest wind comes through the Gurbantunggut desert) [48]. Relevant studies have shown that the dust storms in Xinjiang usually occurred in spring and summer [49,50], and affect a large region downwind, such as the glaciers in the Tibetan Plateau [51], Eastern Asia [52], and across the Pacific, even influencing the air quality over North America [53]. The highest PM concentrations were recorded in cities surrounding the Taklimakan Desert during the spring season and the highest PM_2.5_/PM_10_ ratio was recorded during the winter, indicating the influence of anthropogenic emissions in winter [50]. However, the PM_2.5_/PM_10_ ratio maintained around 0.1 during the two events occurred from November 25^th^ to December 2^nd^ (Fig 8), combined with the characteristics of the weather at the time of the pollution events that the local air humidity increased significantly, accompanied by precipitation and strong winds, indicated that the pollutants of the two events dominated by sandstorm. Backward trajectory analysis showed that the airflow from Central Asia Kazakhstan carries a large amount of dust pollutants (the concentration of PM_10_ reached 2031.76 μg·m^-3^, Table 4) when passing through Gurbantunggut Desert, which is blocked by Tianshan Mountains bordered in the east, west, and the south of Urumqi, and forms sandstorms under the influence of winter downward flow, strengthened the concentrations of the PM_10_, reached 4578 μg·m^-3^.

Examining the source of atmospheric pollutants is help for a better understanding of the formation mechanisms of pollution events [54–56]. Relevant studies have shown that air masses from different regions contribute differently to air pollution [57–59]. An analysis of the trajectories of PM_2.5_ transport in the Yangtze River Delta found that most of the heavily polluted days belonged to shorter trajectory groups and were controlled by high-pressure [60]. In the Lanzhou city, which is locates in Northwest inland region of China, backward trajectory analysis revealed that airflow from sand sources was prone to cause PM_10_ pollution events [61]. The present study found that although the long-distance airflow trajectories from Central Asia was fewer, the sand and dust carried by these long-distance airflow trajectories had a higher contribution to air pollution in Urumqi. In contrast, although there were more short-distance trajectories, due to relatively lower of concentrations of pollutants, they contributed lesser than long-distance ones.

## 5 Conclusions

At present the issue of air quality has become a focus for city with the rapid development of economic and urbanization. In this paper, Urumqi city in Xinjiang province of China is selected as a study area to analysis the characteristics and sources of atmospheric pollutants (PM_2.5_, PM_10_, SO_2_, NO_2_, CO and O_3_8h_) based on conventional statistical methods and backward trajectory analysis. The results showed: (1) The annual average concentrations of major atmospheric pollutants showed different decline trend during the period from 2014 to 2018; the intra-annual variations of PM_2.5_, PM_10_, SO_2_, CO and NO_2_ were similar with lowest and highest value appeared in summer and winter, respectively; diametrically, the concentrations change of O_3_8h_ reached highest in summer and lowest in winter. (2) AQI is higher from November to March and lower from April to October with values varies between 50 and 100. (3) The temperature obtained relatively higher correlation relationship with air quality compare with humidity, air pressure, and wind speed. (4) Airflows arrived to Urumqi can been clustered into four categories using backward trajectory analysis, and the one from Central Asia contributed the mostly to the concentration of PM_2.5_ and PM_10_.

## Supporting information

S1 Dataset (ZIP)
